# 

**Published:** 1887-10-29

**Authors:** 


### Notice to Advertisers.

The scale of charges for small prepaid Advertisements—Situations Wanted, Situations Vacant, etc., etc., is as follows:—

| | s. | d. |
|---|---|---|
| One insertion of three lines | 1 | 0 |
| Three „ „ „ | 2 | 6 |
| Per line afterwards | 0 | 6 |

*A line averages ten words.*

All copy for Advertisements must be sent to A. P. Watt, 2, Paternoster Square, E.C., by first post on the Tuesday *preceding* publication.

### NOTICE TO CORRESPONDENTS.

All correspondence must be written on one side of the paper only.
We cannot undertake to return MSS. not used.
**Communications**, whether intended for insertion or for private information, must be authenticated by the names and addresses of the writers, not necessarily for publication.
**Local papers** containing reports or news paragraphs **should be marked.**
It is especially requested that early intelligence of **local events of interest**, or which it may be thought desirable to bring under notice, may be sent direct to the Editor.
Communications respecting editorial matters should be addressed to the Editor, Norfolk House, Norfolk Street, Strand, London, W.C.

*** Amusements with Prizes for all Ages will be found on page iv of this week's issue.

### Hospitals, Doctors, Treatment, and Attendance.

### THE IN- AND OUT-PATIENTS' HOSPITAL DIARY.

This Diary is so arranged as to make some portion of it appear every week, and the whole in each monthly part. It has been very difficult to compile, and corrections will at all times be gratefully received. It has been suggested that similar information about the larger Provincial and Scotch Hospitals would be useful to many readers. We should be glad to hear if this is felt to be the case.

### PARALYSIS, EPILEPSY, AND DISEASES OF THE NERVOUS SYSTEM GENERALLY.—*Continued.*

| Hospitals and Terms of Admission. | Physicians, Surgeons, and Medical Officers, with Days and Hours of Attendance. |
|---|---|
| **National Hosp. for the Paralysed and Epileptic**, Queen Square, Bloomsbury, W.C. Sec. and Director, Mr. B. B. Rawlings. Most of the beds are reserved free to the necessitous poor; the others are for patients who contribute a Guinea a week | *In-Physicians*, Drs. C. B. Radcliffe, M.; J. S. Ramskill, Tu.; T. Buzzard, W.; J. H. Jackson, F. Hour, 2.30. *In-Surgeons*, Messrs. W. Adams, M. 3; V. Horsley, F. 2.30. *Out-Physicians*, Drs. W. R. Gowers, M.; H. C. Bastian, Tu.; J. A. Ormerod, Tu. W.; T. Buzzard, W.; D. Ferrier, F.; C. E. Beevor, M. F. Hour, 2.30 |
| **National Hospital for Diseases of the Heart and Paralysis**, 32, Soho Sq., W. Sec., Capt. F. Handley. In-patients require recommendation of Life Governor. | *In-Physicians*, Drs. A. Duncan, J. Murray, Stretch Dowse, M. W. Th. *In-Surgeon*, Mr. Graham Bennett, Th. *Out-Physicians*, Drs. R. Verley, J. Murray, Stretch Dowse, Tu. Th. F. 3; M. W. 3 and 8. *Out-Surgeon*, Mr. G. Bennett, Th. 3 |
| **West End Hosp. for Diseases of the Nervous System**, 73, Welbeck St., W. Hon. Sec., Mr. H. A. Dowell. Children only as in-patients. Out-patients by letter or payment. | *In- and Out-Physicians*, Drs. H. Tibbits, M. Th. 1.30; Stretch Dowse, Tu. W. 1.30; S. H. Armitage, W. Tu. F. 6. *In- and Out-Surgeon*, Mr. Benson |

### DISEASES OF THE SKIN.

| | |
|---|---|
| **Charing Cross Hosp.** West Strand, W.C. | *Physician*, Dr. Sangster, M. 2 |
| **Guy's Hosp.**, St. Thomas Street, S.E. | *Physicians*, Drs. Hale-White, Pye-Smith, Tu. 12 |
| **Hospital for Diseases of the Skin**, 52, Stamford St., S.E. Sec., Mr. S. Hayman. Free to the necessitous; also by payment | *Physician*, Dr. F. J. Payne. *Surgeons*, Messrs. J. Hutchinson, Waren Tay, and Wyndham Cottle. (Out-patients seen M. W. 3, Tu. Th. F. 2) |
| **King's College Hosp.**, Portugal Street, W.C. | *Physician*, Dr. Duffin, F. 1 |
| **London Hospital**, Whitechapel Road, E. | *Physician*, Dr. S. Mackenzie, Th. 9 |
| **London Skin Hosp.**, 47, Cranbourn St., Leicester Sq. | Mr. J. Startin, M. and Th. at 2, Tu. evening at 7; Mr. Walter Pocock, Tu. and F. at 2, W. evening at 7; Dr. Barry, W. and S. at 2, F. evening at 7 |
| **Middlesex Hospital**, Berners Street, W. | *Physician*, Dr. R. Liveing, Tu. 4 |
| **North-West London Hospital**, 18, Kentish Town Rd., N.W. | *Physician*, Dr. J. Stowers, Tu. 1.30 |
| **Paddington Green Children's Hosp.**, Paddington | *Physician*, Dr. T. Colcott Fox, S. 9.15 |
| **St. Bartholomew's Hospital**, Smithfield, E.C. | *Surgeon*, Mr. H. Cripps, F. 1.30 |
| **St. George's Hosp.**, Hyde Park Corner, S.W. | *Physician*, Dr. Cavafy, W. 1.30 |
| **St. John's Hosp. for Diseases of the Skin**, 45, Leicester Sq., W.C. Branch, Markham Square, Chelsea, S.W. Sec., Mr. St. Vincent Mercier. In-patients by letter; out-patients free or by small payment | *Physicians*, Drs. Bourns, Campbell, Dow. Harries, Robinson. Daily 2 and 7. Markham Sq. Branch, M. 10 and Th. 10 and 7. *Surgeon*, Mr. J. Milton, Tu. |
| **St. Mary's Hosp.**, Cambridge Place, Paddington, W. | *Surgeon*, Mr. Morris, M. Th. 9.30 |
| **St. Thomas's Hospital**, Westminster Bridge Road | *Physician*, Dr. Payne, W. 1.30 |
| **University College Hospital**, Gower Street, W.C. | *Physician*, Dr. Crocker, W. 1.30, S. 9.15 |
| **Westminster Hospital**, Broad Sanctuary, S.W. | *Physician*, Dr. T. C. Fox, W. 1.30 |

### STONE AND OTHER DISEASES OF URINARY ORGANS.

| | |
|---|---|
| **London Hospital**, Whitechapel Road, E. | Daily, 1 30. *See* General Hospitals |
| **St. Peter's Hospital for Stone and Urinary Diseases**, Henrietta Street, W.C. Sec., Mr. W. E. Scott. Admission free. Only women and children are seen on Fridays. Operation day, Wednesday | *In-Surgeons*, Messrs. W. J. Coulson, F. R. Heycock. *Out-Surgeons*, Messrs. E. H. Fenwick, M. 2, S. 4 to 7; F. S. Edwards, M. 5 to 7, Th. 2; Heycock, Tu. 2, W. 5 to 7, F. 2 |

### DISEASES AND IRREGULARITIES OF THE TEETH.

| Hospitals and Terms of Admission. | Physicians, Surgeons, and Medical Officers, with Days and Hours of Attendance. |
|---|---|
| **Belgrave Hosp. for Children**, 77 & 79 Gloucester St., Pimlico | *Surgeon*, Mr. G. W. Payne, W. 9 |
| **Charing Cross Hospital**, West Strand, W C. | *Surgeon*, Mr. Fairbank, M. W. F. 9 |
| **Dental Hospital of London**, Leicester Sq., W.C. Sec., Mr. J. F. Pink. Poor free. For special operation a Governor's letter is required | *Surgeons*, Messrs. S. Bennett, Canton, Gregson, Hepburn, Hern, Matheson, Parkinson, Read, Rogers, Truman, Underwood, Woodhouse. Daily, 9 to 11. |
| **Evelina Hosp. for Sick Children**, Southwark Brdg. Rd. | *Surgeon*, Mr. R. Denison Pedley, Tu. 9 |
| **German Hospital**, Dalston lane, Dalston, E. | *Surgeons*, Messrs. A. P. Reboul, C. West, Th. 10 to 11 |
| **Great Northern Central Hosp.**, Caledonian Rd, N. | *Surgeon*, Mr. E. Keen, W. 1.30 |
| **Guy's Hosp.**, St. Thomas Street, S.E. | *Surgeons*, Messrs. Moon, Pedley, Th. F. 12.30 |
| **Hospital for Consumption**, Brompton, S.W. | *Surgeon*, Mr. C. J. Noble, W., when wanted |
| **Hospital for Sick Children**, Gt. Ormond St., W.C. | *Surgeon*, Mr. Cartwright, W. 9 |
| **King's College Hosp.**, Portugal Street, W.C. | *Surgeon*, Mr. Cartwright, Tu. F. 10 |
| **London Hospital**, Whitechapel Road, E. | *Surgeon*, Mr. A. W. Barrett, Tu. 9 |
| **London Temperance Hosp.**, Hampstead Rd, N.W. | *Surgeon*, Mr. Adolphus Alexander, M. 2 |
| **Metropolitan Free Hospital**, Gray's Inn Rd., W.C. | *Surgeon*, Mr. G. Curnock, Tu. Th. S. 9 |
| **Middlesex Hosp.**, Berners Street, W. | *Surgeons*, Messrs. Bennett, M. 9.30; W. 9; Hern, W. 9, F. 9.30 |
| **National Dental Hosp.**, 149, Gt. Portland Street, W. Sec., Mr. A. G. Klugh. The poor, and all urgent cases admitted free; others by Subscriber's letter | *Surgeons*, Messrs. H. Weiss, W. Weiss, M.; A. Smith, G. Bradshaw, Tu.; G. A. Williams, M. Davis, W.; A. F. Canton, H. G. Read, Th.; T. Gaddes, W. S. Thomson, F.; H. Rose, W. R. Humby, S. Hours 9 to 11 |
| **National Orthopædic Hosp.**, 234, Great Portland Street, W. | *Surgeon*, Mr. H. Harris |
| **New Hosp. for Women**, 222, Marylebone rd., W. | *Surgeon*, Mr. H. Apperley |
| **North-West London Hosp.**, 18, Kentish Town rd., N.W. | *Surgeon*, Mr. W. A. Maggs, F. 9 |
| **Paddington Green Children's Hosp.**, Paddington | *Surgeon*, Mr. C. Vincent Cotterell, W. 9 |
| **Royal Free Hosp.**, Gray's Inn Road, W.C. | *Surgeon*, Mr. H. Harris, M. Th. |
| **Royal Hosp. for Children and Women**, Waterloo Bridge Road, S.E. | *Surgeon*, Mr. Whitehouse, F. 1.30 |
| **St. Bartholomew's Hospital**, Smithfield, E.C. | *Surgeons*, Messrs. Ewbank, Tu.; Paterson, F.; Ackery, F.; Mackrett, Tu. Hour, 9 |
| **St. George's Hosp.**, Hyde Park Corner, S.W. | *Surgeon*, Mr. Winterbottom, Tu. S. 9 |
| **St. Mary's Hosp.**, Cambridge Place, Paddington | *Surgeon*, Mr. H. Hayward, W. S. 9.30 |
| **St. Thomas's Hospital**, Westminster Bridge Rd., S.E. | *Surgeon*, Mr. Truman, Tu. F. 10 |
| **University College Hospital**, Gower Street, W.C. | *Surgeon*, Mr. S. J. Hutchinson, W. 9.30 |
| **Victoria Hosp. for Children**, Chelsea, S.W. | *Surgeon*, Mr. F. Fox, S. 9.0 |
| **West London Hospital**, Hammersmith Road, W. | *Surgeon*, Mr. H. L. Albert, Tu. F. 9.30 |
| **Westminster Hospital**, Broad Sanctuary, S.W. | *Surgeons*, Messrs. J. Walker and M. A. Smale W. S. 9.15 |

### THROAT DISEASES.

| | |
|---|---|
| **Central London Throat and Ear Hosp.**, Gray's Inn Road, W.C. | *Surgeons. See* "Ear Cases." M. W. Th. S. 2.30; Tu. F. 5 |
| **Great Northern Central Hosp.**, Caledonian Rd, N. | *Surgeon*, Mr. Spencer Watson, W. 2 |
| **Guy's Hosp.**, St. Thomas Street, S.E. | *Surgeon*, Mr. Symonds, Th. 1 |
| **Hospital for Diseases of the Throat and Chest**, 32, Golden Sq. Hon. Sec., Mr. G. C. Witherby. Poor free; others by small payment | *Physicians*, Drs. Wolfenden, Tu. F. 2.30; Macdonald, Tu. F. 6.30; Bond, W. S. 2.30. *Surgeon*, Mr. T Mark Hovell, M. Th. 2.30 |

### AMUSEMENTS WITH PRIZES, FOR ALL AGES.

**Rules.**—All answers must be addressed to the PRIZE EDITOR, THE HOSPITAL, Norfolk House, Norfolk Street, Strand, W.C., not later than the *first* post on Friday next. The full name and address must in all cases be given, but a *nom de plume* may be added for publication. Only one side of the paper must be written on. No one will be permitted to win the Monthly or Quarterly Prizes twice in any one year, the annual prizes being offered especially to prevent this.

### FOR MEN AND WOMEN.

### Third Quarterly Competition.

RESULTS OF LAST WEEK.

**Answers.**

No. 25. DOUBLE ACROSTIC.

F anati C
A s H
R ogu E
A nthe M
D israel I
A lbatros S
Y ach T

Correct answers have been received from Toby, Esau, Aralc, Reldas, Gretta, Lady Clara, Ellice, Perseverance, Condorcet, Boss, Mater, Brisbane, Brownie.

No. 26. ENIGMA.
Hair.

Correct answers have been received from Toby, Aralc, K. M., Reldas, Gretta, Perseverance, Condorcet, Boss, Mater, Brisbane, Brownie.

**Results of Third Quarterly Competition.**

MATER and CONDORCET having each gained 23 marks, the highest number scored, we again propose either (1) to divide the prize, or (2) to set a test acrostic to decide the tie. We shall be glad to hear from MATER and CONDORCET which course they prefer.

**Notices to Correspondents.**

GRETTA.—Your answer was entirely wrong.

### THE CHILDREN'S CORNER.

PRIZES.
FOR MOST CORRECT ANSWERS TO PUZZLES.
One Pound per Month.
FOR BEST LITTLE LETTERS.
Ten Shillings per Quarter.
A PRIZE OF THREE GUINEAS
to the Competitor who shall have sent in the largest number of correct or best answers during the twelve months.

**Puzzles—Fifth Week.**

No. 16. MISSING LETTER PUZZLE.

T×e×a×w×s×o×g×h×w×n×w×s×o×d ×h×m×n×t×e×w×s×n×i×m×n×o×d H×s×i×h×r×d×h×e×a×d×r×s×e× g×e× ×e×m×d×o×a×e×n×w×a×e×t×r×a×

No. 17. GEOGRAPHICAL DIAMOND PUZZLE.

    *          1. A river.
  * * *        2. A period of time.
* * * * *      3. A naval commander.
  * * *        4. A verb.
    *          5. A vowel.

No. 18. FRENCH PUZZLE. Make a French sentence of the following:

L n é né o p y

No. 19. HISTORICAL RIDDLE-ME-REE.

My first is in coffee, but not in tea,
My second in water, but not in sea.
My third is in flower, but not in leaf,
My fourth is in lamb, but not in beef.
My fifth is in cow, but not in bull,
My sixth is in empty, but not in full.
My seventh is in heel, but not in toe,
My eighth is in foil, but not in foe.

**Letters—Fifth Week.**

Next week tell me all you can of interest about *Lambeth Palace*.

**Results of Third Week—Puzzles.**

No. 9. NATURAL HISTORY ACROSTIC.

S ilkwor M

P otoro O

I chneumo N

D uc K

E rmin E

R a Y

No. 10. GEOGRAPHICAL CHARADE. King-s ton=Kingston.

No. 11. A troublesome insect—wasp, transposed, gives paws. Take away p, and you find was. Reverse, and you get saw, a useful instrument.

RESULTS.

*All right*—Skye, Ossian, A. C. K., Toby, Tim, Paddy, G. B. P.; *Two right*—Poodle, Jolly Sailor, Judy, Joe, Punch, Gem, Jerusalem, Pickles, Joe Pena, Mount Edgcumbe, Alsie, Scrooge, Pepita, Spider, A. G. S. McCulloch; *One right*—Princess Ida, Fern.

**Westminster Abbey.**

On the whole, the girls have sent in the best letters this week on *Westminster Abbey*. Princess Ida writes a very good letter, and relates graphically the old legend of the supernatural consecration of King Sebert's church. One correspondent is rather hazy as to dates, and I commend to his notice the following little letter

BY GEM.

Westminster Abbey was founded, or rather refounded by Edward the Confessor, on the supposed site of King Sebert's Church. Edward the Confessor finished it just before his death, and it was dedicated in 1065 to St. Peter. William the Conqueror was crowned in it in 1066, when there was a mistake made by the Norman soldiers, who thought the Saxon shouts within was a rebellion, and immediately set upon the people, and burnt some houses. The riot was only stopped by King William himself. Almost all the kings and queens of England have been crowned in the Abbey. Henry VII added a beautiful chapel, in which he himself was buried. Once, in Charles I's civil wars, it was made into a barrack for soldiers in 1643. In 1837 our own gracious Queen was crowned there; in 1865 the 800th anniversary of its dedication was celebrated; and on June 21st, 1887, the Jubilee of Queen Victoria was celebrated here, before thousands of spectators. God save the Queen.

BY DAISY.

Westminster Abbey, the building of which all Englishmen are proud, was, we are told, originally a Benedictine monastery, the "Minster West" of St. Paul's, London. There are nine chapels altogether, bearing different names; of which the most interesting is the chapel of Edward the Confessor, not only because his shrine is there, which was erected in the reign of Henry III, and which was at one time supposed to have worked miracles, but also because of the coronation chair, which contains the stone of Scone, on which the Scottish kings were crowned, and which some say was brought over in distant times from Palestine, and found its way into Scotland through Ireland; but this is tradition only. It was on this chair of hoary age that Queen Victoria sat on the day of her Jubilee. The next great point of interest to us as a nation is the "Poets' Corner," where Chaucer lies, and Spenser, and Shakespeare, and many, many others. Then there is the Chapter House, which Dean Stanley tells us was the "first home of the House of Commons," built by Henry III in 1250. Its roof is held up by a central pier, used in the monks' time as a whipping-post for them, poor things. Then there is the Jerusalem Chamber, where Henry IV died, remarked on by Shakespeare; and where the New Testament was revised not long ago. This is all I can think of about Westminster Abbey, except that I should like to see it very much, and I hope I shall some day whenever I go up to London.

The rest of the letters received are fairly good, but would have been better had they contained more incident—some correspondents getting no further than William the Conqueror.

Three marks each:—Princess Ida, Daisy, Gem, Ossian. Two marks each—Skye, Twaddle, Poodle. One mark each—Fern, Spider.

**Notices to Correspondents.**

SPIDER.—Your name was inadvertently omitted last week amongst those who received three marks for puzzles and one mark for letter.

### THEATRES AND CONCERTS.

**The Red Lamp.**

NOW that the long winter evenings are come round again people are turning anxiously to see what is going on at the theatres. The result is readily noticed by the fact that nearly all the many theatres in London are once more open after the summer vacation, and doing good business. This success is well deserved, for in their various styles all have an excellent programme, and "something worth seeing." Amongst so abundant a provision of good things it was difficult to make a choice of the best theatre to begin with, but as often happens, accident decided the matter for us.

The Strand is and always has been the centre of theatres, but on the way to the Strand from the West-end one generally goes down the Haymarket, and in the Haymarket may now be seen burning a bright red lamp. Its attractions are great, so great indeed that curiosity is naturally aroused to see what is going on in the historic theatre. The lamp has been burning more than one hundred nights, and still burns, to the delight of as large audiences as ever. The play to which the above title has been given, written by W. O. Tristram, was first put on the stage o: the Comedy Theatre in March last, and after a short vacation has been re-lighted in the old Haymarket Theatre, where it rivals in popularity its predecessor, *Jim the Penman*. The theatre has been re-seated and re-arranged, and, in answer to the popular demand, Mr. Tree has in name restored the pit, though not in its old place. Comfortable seats are provided for the great two-and-sixpenny paying public, and they get good money's worth. The house is very cosy, and what is well to note in these times of fire scares, well provided with exits, and perfectly safe.

The *Red Lamp* has been written to throw light upon the cruelty with which Nihilism is mercilessly stamped out in Russia, and the extent to which its principles have penetrated society, even the most princely houses being divided against themselves, some members struggling with the greatest energy to put down and expose plots in which those nearest and dearest to them may be, without their knowledge, irretrievably implicated. Mr. Tree has reserved to himself the part of Demetrius, which will be remembered as one of the best representations of this clever artist. It is well suited to his particular talents, and has been much improved by him since its first presentation. As Paul Macari in *Called Back* he was very good, and now as Chief of the all-powerful Russian police he holds his audience. It is delightful to hear the *sang froid* with which he sometimes raises a laugh by his two catch-words, improved by a nasal chuckle. Invited to sit, he replies, in his truly comical manner, "No, thanks, I don't sit—a mere whim—a mere whim." Invited to smoke, "No, thanks, I don't smoke—a mere whim." Mrs. Tree fills the part of Princess Claudia Morakoff, the scourge of Nihilism, with a most charming ability. Her place was originally filled by Lady Monkton, of *Jim the Penman* fame. As General Morakoff Brookfield acts with success, and shows an unrelenting vigour in hunting down the Nihilists, though he allows himself to be rather too easily "pumped" by his wife as to projected raids for the success thereof. Two of the cleverest pieces of acting in the piece are the Yankee journalist, a very wide-awake gentleman who thoroughly enlists the sympathies of the house as presented by Mr. Charles Sugden; and the smartness of Miss Filippi, a warning to all ladies who are inclined to put too much trust in French maids. The piece begins at 8 o'clock, is carried through with much spirit till 10 p.m., and certainly should be seen.

